# Quantitative expression analysis and prognostic significance of L-DOPA decarboxylase in colorectal adenocarcinoma

**DOI:** 10.1038/sj.bjc.6605654

**Published:** 2010-04-27

**Authors:** C K Kontos, I N Papadopoulos, E G Fragoulis, A Scorilas

**Affiliations:** 1 Department of Biochemistry and Molecular Biology, Faculty of Biology, University of Athens, Athens GR-15701, Greece; 2 Fourth Surgery Department, University General Hospital ‘Attikon’, 1 Rimini Street, Athens GR-12462, Greece

**Keywords:** colorectal adenocarcinoma, L-DOPA decarboxylase, *DDC*, real-time PCR, tumour biomarkers

## Abstract

**Background:**

L-DOPA decarboxylase (DDC) is an enzyme that catalyses, mainly, the decarboxylation of L-DOPA to dopamine and was found to be involved in many malignancies. The aim of this study was to investigate the mRNA expression levels of the *DDC* gene and to evaluate its clinical utility in tissues with colorectal adenocarcinoma.

**Methods:**

Total RNA was isolated from colorectal adenocarcinoma tissues of 95 patients. After having tested RNA quality, we prepared cDNA by reverse transcription. Highly sensitive quantitative real-time PCR method for *DDC* mRNA quantification was developed using the SYBR Green chemistry. *GAPDH* served as a housekeeping gene. Relative quantification analysis was performed using the comparative C_T_ method (2^−ΔΔC_T_^).

**Results:**

*DDC* mRNA expression varied remarkably among colorectal tumours examined in this study. High *DDC* mRNA expression levels were found in well-differentiated and Dukes’ stage A and B tumours. Kaplan–Meier survival curves showed that patients with *DDC*-positive tumours have significantly longer disease-free survival (*P*=0.009) and overall survival (*P*=0.027). In Cox regression analysis of the entire cohort of patients, negative *DDC* proved to be a significant predictor of reduced disease-free (*P*=0.021) and overall survival (*P*=0.047).

**Conclusions:**

The results of the study suggest that *DDC* mRNA expression may be regarded as a novel potential tissue biomarker in colorectal adenocarcinoma.

Colorectal carcinoma (CRC) is the third most common malignant tumour and the fourth most common cause of cancer death in the world (Parkin *et al*, 2001a; Jemal *et al*, 2009). The prognosis of patients with CRC largely depends on the degree of penetration of the tumour through the bowel wall, the nodal status, and the presence or absence of distal metastases (Steinberg *et al*, 1986). These three characteristics are the key features of all clinical staging systems developed for this disease (Compton and Greene, 2004).

In spite of the fact that clinicopathological staging separates patients with CRC into groups with distinct outcomes, it provides little information about response to treatment in individual patients (Walther *et al*, 2009). In an attempt to refine prognostication and predict the benefit derived from systemic treatment, several protein and genetic markers have been evaluated in patients with CRC, including allelic loss of chromosome 18q (Jen *et al*, 1994; Martinez-Lopez *et al*, 1998; Ogunbiyi *et al*, 1998; Popat and Houlston, 2005), absence of the deleted in colorectal carcinoma (DCC) protein (Shibata *et al*, 1996; Reymond *et al*, 1998; Popat and Houlston, 2005), decreased *SMAD4* mRNA expression (Boulay *et al*, 2002; Alazzouzi *et al*, 2005), expression and/or abnormalities of cytoplasmic oncoprotein p53 (TP53) (Sun *et al*, 1992; Munro *et al*, 2005; Russo *et al*, 2005), protein levels and/or gene haplotype of thymidylate synthetase (TYMS) (Popat *et al*, 2004; Suh *et al*, 2005; Tsourouflis *et al*, 2008), microsatellite instability (MSI) (Gryfe *et al*, 2000; Popat *et al*, 2005), and chromosomal instability (CIN) (Walther *et al*, 2008). Nevertheless, none of these biomarkers has been prospectively validated and established so far in clinical practice. Hence, the identification of new, reliable prognostic and predictive biomarkers that will contribute utmost to clinical decision-making, remains an important research topic (Locker *et al*, 2006; Walther *et al*, 2009).

L-DOPA decarboxylase (DDC) is a pyridoxal 5-phosphate (PLP)-dependent enzyme that catalyses the decarboxylation of 3,4-dihydroxy-L-phenylalanine (L-DOPA) to dopamine (DA) and 5-hydroxy-L-tryptophan (S-HTP) to serotonin (5-HT) (Christenson *et al*, 1972). DDC is expressed in the central nervous system as well as in peripheral organs, such as the liver, kidney, pancreas and placenta (Lindstrom and Sehlin, 1983; Maneckjee and Baylin, 1983; Ichinose *et al*, 1989; Mappouras *et al*, 1990; Siaterli *et al*, 2003). Moreover, enzymatically active DDC was recently found in human leukocytes as well as in the histiocytic lymphoma cell line U-937, thus suggesting a cross-talk between the nervous and the immune systems and raising new questions about the regulatory role of DDC in immune responses (Kokkinou *et al*, 2009a, 2009b). Recently, two endogenous inhibitors of the enzymatic activity of DDC have been identified and purified, one from human serum and another from the membrane fraction of human placental tissue, yet their biological significance remains unexplored (Vassiliou *et al*, 2005, 2009).

The structure of the human *DDC* gene has been fully determined. The single-copy gene encoding *DDC* maps to chromosome 7p12.2, close to the epidermal growth factor receptor (EGFR) gene, and is composed of 15 exons spanning a genomic region of more than 85 kb (Ichinose *et al*, 1989; Sumi-Ichinose *et al*, 1992). Furthermore, two other *DDC* mRNA transcripts encoding distinct DDC protein isoforms as well as alternative splicing in 5′-untranslated region have been identified and characterised (Krieger *et al*, 1991; Ichinose *et al*, 1992; O’Malley *et al*, 1995; Vassilacopoulou *et al*, 2004).

It is worth mentioning that DDC is regarded as a general biomarker for neuroendocrine tumours (Gazdar *et al*, 1988; Gilbert *et al*, 1995; Ippolito *et al*, 2005). High *DDC* mRNA expression has been noticed in small-cell lung carcinoma (SCLC), neuroblastoma, and pheochromocytoma (Jensen *et al*, 1990; Gilbert *et al*, 1999; Uccella *et al*, 2006). Moreover, it has been suggested that *DDC* mRNA levels could be a potential biomarker for the detection of minimal residual disease in patients with neuroblastoma and for the discrimination of neuroblastoma from other small round-cell tumours of childhood (Gilbert *et al*, 1999; Bozzi *et al*, 2004).

Recent studies revealed that DDC is also implicated in prostate cancer neuroendocrine differentiation, accounting for abnormal prostate cell proliferation and differentiation (Wafa *et al*, 2007), as it is an androgen receptor (AR) coregulator protein acting at the cytoplasmic level to enhance AR activity and to differentially modulate AR-regulated genes (Wafa *et al*, 2003; Margiotti *et al*, 2007). Not surprisingly, *DDC* gene expression at the mRNA level has been proposed as a novel tissue biomarker in prostate cancer (Avgeris *et al*, 2008). *DDC* is also overexpressed in peritoneal dissemination of gastric carcinoma, and its quantification has been shown to be reliable and effective for the selection of patients for adjuvant intraperitoneal chemotherapy, aiming at preventing peritoneal recurrence (Sakakura *et al*, 2004). Still, the role of DDC in CRC remains unclear.

The above data encouraged us to analyse the *DDC* mRNA expression in colorectal adenocarcinoma specimens, developing an ultra-sensitive and highly accurate quantitative real-time PCR methodology using the SYBR Green chemistry, and to examine its potential prognostic significance and clinical application as a novel molecular tissue biomarker for colorectal adenocarcinoma.

## Materials and methods

### Tissue samples and RNA isolation

Included in this study were tumour specimens from 95 patients having undergone surgical treatment for primary colorectal adenocarcinoma between 2000 and 2003. The selection criteria for the specimens included the availability of sufficient tissue mass for RNA extraction and assay. Tumour tissues had been frozen in liquid nitrogen immediately after their surgical resection.

Tissue specimens were pulverised and then dissolved in TRI Reagent (Ambion (Europe) Ltd., Huntingdon, UK). Following the manufacturer's instructions, we extracted and diluted total RNA in an RNA Storage Solution (Ambion Ltd), and stored it at −80°C until use.

Patient age ranged from 35 to 88 years with a mean±s.e. of 67.3±1.01. Other patients’ characteristics and stage of tumours are shown in Tables 1,2,3. Follow-up information was available for 72 patients and included survival status (alive or deceased) and disease status (disease-free or recurrence/metastasis) along with the dates of the events and cause of death.

The study was performed with respect to the ethical standards of the 1975 Declaration of Helsinki Principles, as revised in 1996, and has been approved by the ethics committee of the University General Hospital ‘Attikon’.

### cDNA synthesis

First-strand cDNA was produced from total RNA by using an RNA PCR Kit Version 3.0 (TaKaRa Bio Inc., Tokyo, Japan), according to the manufacturer's instructions. The reaction mixture contained 2 *μ*g of total RNA diluted in 11 *μ*l of diethylpyrocarbonate (DEPC)-treated water, 2.5 pmol Oligo dT-Adaptor primer, 2 *μ*l RT Buffer (10 × , 100 mM Tris-HCl (pH=8.3), 500 mM KCl), 4 *μ*l 5 mM MgCl_2_, 2 *μ*l 10 mM dNTP mix, 20 U RNase inhibitor (Rnase Inhibitor; 40 U *μ*l^−1^; TaKaRa Bio Inc.) and 1.25 U reverse transcriptase (AMV Reverse Transcriptase XL; 5 U *μ*l^−1^; TaKaRa Bio Inc.). The final reaction volume was 20 *μ*l. The reaction mixture was incubated at 30°C for 10 min, 60°C for 30 min, and the reaction was terminated by heating the mixture at 99°C for 5 min and cooling it at 5°C for 5 min.

### Real-time quantitative PCR

**Figure 1:**
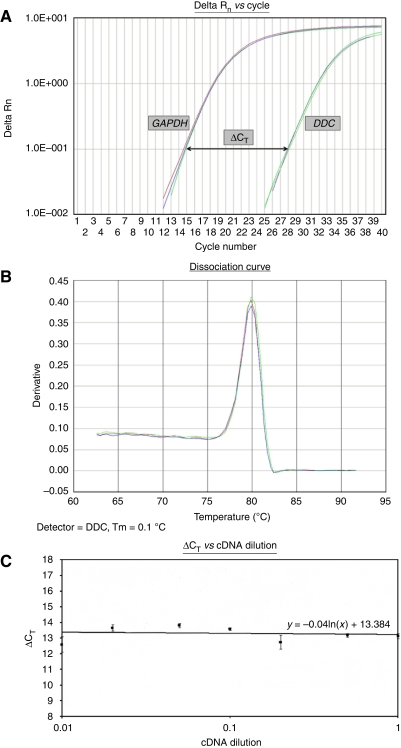
Real-time PCR quantification of *DDC* gene expression in colon tissues. (**A**) Amplification plot of *DDC* and *GAPDH* cDNAs, showing ΔR_n_ plotted *vs* cycle number. *DDC* mRNA expression was detected by real-time quantitative PCR, using the SYBR Green chemistry, while *GAPDH* served as a reference gene. Calculations were made with the use of the comparative C_T_ (2^−ΔΔC_T_^) method. (**B**) Dissociation curves of the *DDC* amplicon, showing the specificity of primers used for the real-time PCR amplification and quantification of *DDC*. Neither primer-dimers nor other non-specific products were observed after melting of the PCR products. (**C**) Validation of the comparative C_T_ (2^−ΔΔC_T_^) method. The efficiency of the amplification of the target gene (*DDC*) and internal control (*GAPDH*) was examined by means of real-time PCR and SYBR Green detection. With the use of reverse transcriptase, cDNA was synthesised from 2 *μ*g total RNA isolated from human MCF7 cells. Serial dilutions of cDNA over a 100-fold range were amplified by real-time PCR using gene-specific primers. The most concentrated sample contained cDNA derived from 100 ng of total RNA. The ΔC_T_ (C_T,*DDC*_–C_T,*GAPDH*_) was calculated for each cDNA dilution and plotted *vs* it. All data were fit using least-square linear regression analysis. The absolute value of the slope of the resulting plot is almost equal to zero, which indicates that the amplification efficiencies for both genes are similar.

Quantitative real-time PCR was performed using the SYBR Green chemistry in a 7500 Real-Time PCR System (Applied Biosystems, Foster City, CA, USA) (Figure 1A). On the basis of the information of the *DDC* and *GAPDH* cDNA sequences, two pairs of gene-specific primers were designed. The reaction mixture contained 50 ng of cDNA diluted in 2.5 *μ*l of DEPC-treated water, 5 *μ*l *Power* SYBR Green PCR Master Mix (2 × ) (Applied Biosystems), and 2 *μ*l of gene-specific primers (final concentration, 50 nM each), in a final reaction volume of 10 *μ*l. The *DDC* real-time PCR primers were 5′-GAACAGACTTAACGGGAGCCTTT-3′ and 5′-AATGCCGGTAGTCAGTGATAAGC-3′, producing a 90-bp PCR amplicon, and the *GAPDH* real-time PCR primers were 5′-ATGGGGAAGGTGAAGGTCG-3′ and 5′-GGGTCATTGATGGCAACAATATC-3′, resulting in a 107-bp PCR amplicon. The cycling conditions were as follow: a denaturation step at 95°C for 10 min, followed by 40 cycles of 95°C for 15 s, 60°C for 60 s, and a final step for the generation of a dissociation curve to distinguish between the main PCR product and primer-dimers (Figure 1B).

Calculations were made with the use of the comparative C_T_ (2^−ΔΔC_T_^) method. *GAPDH* was used as an internal control gene to normalise the PCRs for the amount of RNA added to the reverse transcription reactions, whereas the breast adenocarcinoma epithelial cell line MCF7 was used as a calibrator for making PCRs from distinct runs comparable (Livak and Schmittgen, 2001). ΔΔC_T_ represents the difference between the mean ΔC_T_ value of a colon sample and the mean ΔC_T_ of the calibrator, both calculated after the same PCR run, whereas ΔC_T_ is the difference between the threshold cycle (C_T_) of the target gene (*DDC*) and the C_T_ of the endogenous reference gene (*GAPDH*) of the same sample.

Normalised results were expressed as the ratio of *DDC* mRNA copies to *GAPDH* mRNA copies calculated for each colon tissue sample in relation to the same ratio calculated for MCF7 cells. The normalised (2**^−^**^ΔΔC_T_^) amounts of tissue sample *DDC* mRNA levels were then multiplied with the average ratio of *DDC* mRNA copies to *GAPDH* mRNA copies of MCF7 cells (2^−13.384^), calculated from the intercept of the regression line shown in Figure 1C, thus resulting in comparable results that do not depend on the *DDC* mRNA expression levels of MCF7 cells. Finally, these results were multiplied by 1000, thus yielding c/Kc (*DDC* mRNA copies per 1000 *GAPDH* mRNA copies). Each real-time PCR reaction was performed in triplicate to evaluate the reproducibility of data.

### Statistical analysis

The X-tile algorithm was used to generate an optimal cut-off point for *DDC* (Camp *et al*, 2004), as there are no established cut-off points concerning its expression in colorectal adenocarcinoma. Having corrected for the use of minimum *P*-value statistics, the X-tile software yielded an optimal cut-off of 12.82 c/Kc, equal to the 75th percentile, with a calculated Monte Carlo *P*-value <0.05. According to this cut-off, *DDC* mRNA expression was classified as positive or negative, and associations between *DDC* status and other qualitative clinicopathological parameters were analysed using the *χ*^2^-test or the Fisher's exact test, where appropriate.

Cox proportional hazard regression model was developed to assess the association between the prognostic markers and the relative risks for relapse and death of patients (Cox, 1972). Cox analysis was conducted at both univariate and multivariate levels. Only patients for whom the status of all variables was known were included in the multivariate regression models, which incorporated *DDC* mRNA expression and all other variables for which the patients were characterised. The multivariate models were adjusted for nodal status, histologic tumour grade, and Dukes’ stage (Dukes, 1932; Hamilton and Aaltonen, 2000).

Survival analyses were also performed by constructing Kaplan–Meier disease-free survival (DFS) and overall survival (OS) curves (Kaplan and Meier, 1958). The differences between the curves were evaluated by the log-rank test.

## Results

### Validation of the comparative C_T_ (2^−ΔΔC_T_^) method for *DDC* mRNA quantification

For the ΔΔC_T_ calculation to be valid, the amplification efficiencies of the target and reference genes must be approximately equal (Livak and Schmittgen, 2001). For this purpose, the C_T_ values for *DDC* and *GAPDH*, corresponding to the number of cycles at which the fluorescence emission monitored in real time reached a threshold of 10 times the standard deviation of the mean baseline emission from cycles 3 to 15 (Figure 1A) (Giulietti *et al*, 2001), were measured in serial dilutions of a control cDNA over a 100-fold range, and the ΔC_T_ (namely the difference C_T,*DDC*_–C_T,*GAPDH*_) was plotted *vs* the log cDNA dilution. The absolute value of the slope of the resulting plot is close to zero, which implies similar amplification efficiencies of both amplicons (Figure 1C).

### *DDC* expression status in colorectal adenocarcinoma tissues and its association with patients’ clinicopathological variables

mRNA expression of *DDC* in colorectal adenocarcinoma tissues varied from 0.04 to 91.95 c/Kc (*DDC* mRNA copies per 1000 *GAPDH* mRNA copies) with a mean±s.e. of 9.02±1.51 (Table 1). Table 2 shows the association of *DDC* mRNA expression status of the tumour with various clinicopathological variables. *DDC* values were classified into two categories (positive or negative), as described in the Materials and Methods section. Out of 95 colon adenocarcinomas examined, 24 (25.3%) were classified as positive for *DDC* expression and 71 (74.7%) as negative. *DDC* positivity was found more frequently in well-differentiated tumours, whereas high-grade colorectal tumours were found to be *DDC*-negative (*P*=0.011). Significant associations between *DDC* status and patient age, nodal status, or Dukes’ stage were not observed.

### *DDC* expression status and colorectal adenocarcinoma survival

**Figure 2:**
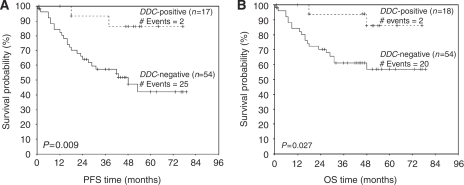
Kaplan–Meier survival curves. Kaplan–Meier curves for disease-free survival (DFS) (**A**) and overall survival (OS) (**B**) of patients with *DDC*-positive and *DDC*-negative colorectal adenocarcinoma. *DDC* expression was found to have a favourable prognostic value for colorectal adenocarcinoma, as patients with *DDC*-positive colorectal adenocarcinoma have significantly longer DFS (*P*=0.009) and OS (*P*=0.027), in comparison with those whose tumours are *DDC*-negative.

Complete follow-up information was available for 72 patients, among whom 27 (38%) had relapsed and 22 (31%) had died. In Cox univariate analysis, histologic tumour grade, and disease Dukes’ stage were significant predictors of DFS and OS, as expected. In addition to these established prognostic factors, *DDC* mRNA expression was found to be an important predictor of DFS and OS (*P*=0.021 and *P*=0.047, respectively). *DDC*-positive patients were found to have a significant lower risk to relapse (HR=0.18) or die (HR=0.23) (Table 3). The Kaplan–Meier survival curves also show that patients with *DDC*-positive tumours have remarkably longer DFS (*P*=0.009) and OS (*P*=0.027), compared to those with *DDC*-negative malignancies (Figure 2). In multivariate analysis, when all parameters were included in the Cox regression model, only Dukes’ stage retained its prognostic significance for DFS and OS of patients with colorectal adenocarcinoma (*P*=0.004 and *P*=0.021, respectively) (Table 3).

## Discussion

Cancer of the colon and rectum is an important public health issue, constituting a major cause of worldwide morbidity and mortality (Pisani *et al*, 2002; Jemal *et al*, 2009). In Europe, CRC is the second most common malignancy among women after breast cancer and the third most frequent in men after prostate and lung cancer. Moreover, CRC is the second most common cause of cancer death for both sexes, accounting for 12% of all tumour-related deaths (Parkin *et al*, 2001b; Ferlay *et al*, 2010). Early diagnosis of CRC and early detection of recurrence after surgery are critical for effective treatment and/or positive clinical outcome. Endoscopic examination of the colon remains the most reliable screening method for this type of malignancy (Newcomb *et al*, 1992; Lieberman *et al*, 2000).

All existing classification systems for CRC distinguish between patients with early-stage CRC and those with very advanced-stage disease; nonetheless, they are less efficient in predicting the prognosis of patients with intermediate levels of tumour burden (McLeod and Murray, 1999). On the basis of studies published over the last few years, the American Society of Clinical Oncology Tumour Marker Panel and the European Group on Tumour Markers have recently suggested that preoperative carcinoembryonic antigen (CEA) levels may be used as an independent prognostic factor, assisting in staging and surgical treatment planning. It should also be noted that CEA is the marker of choice for monitoring the response of metastatic disease to systemic therapy (Duffy *et al*, 2003; Locker *et al*, 2006). However, neither CEA nor any other biomarkers that have been proposed in the past, such as CA19-9, have enough sensitivity for colon cancer detection (van der Schouw *et al*, 1992; Pokorny *et al*, 2000; Locker *et al*, 2006).

L-DOPA decarboxylase is a PLP-dependent enzyme participating in the catecholamine biosynthesis pathway, responsible principally for the synthesis of the key neurotransmitters DA and 5-HT (Christenson *et al*, 1972). Biogenic amines are generally considered to participate in various processes, such as angiogenesis, cell proliferation, differentiation and apoptosis (Berry *et al*, 1996; Medina *et al*, 1999; Lang *et al*, 2005), which implies a potentially significant role of DDC in cancer pathobiology and progression. Interestingly enough, it has recently been shown that catecholamines, including DA itself, inhibit erythrocyte apoptosis by preventing scramblase activation and subsequent phosphatidylserine exposure on the cell membrane (Lang *et al*, 2005), which in turn triggers clearance of apoptotic cells by macrophages.

*DDC* mRNA expression is used for the differential diagnosis of neuroblastoma from other pediatric small round-cell malignancies (Gilbert *et al*, 1999; Bozzi *et al*, 2004). Quantification of *DDC* mRNA expression using real-time RT-PCR has been proposed as a method useful for the prediction of peritoneal recurrence in patients with gastric carcinoma (Sakakura *et al*, 2004). In addition, DDC protein expression is a biomarker of neuroendocrine differentiation in SCLC cells and prostate carcinoma (Gilbert *et al*, 2000; Wafa *et al*, 2007). *DDC* mRNA levels were also found to be particularly elevated in cancerous prostate tissue, in comparison with benign prostate hyperplasia. High-expression levels of *DDC* were found to be associated with more aggressive prostate tumours (Avgeris *et al*, 2008).

Several peripheral cancers are characterised by an extremely high DDC activity, associated with the tumour. This is especially apparent with lung cancers of small-cell origin, although variants showing no protein expression have been observed as well (Berry *et al*, 1996). Remarkable increase in DDC activity, in comparison with normal tissue levels, is also seen in primary intestinal cancer and its related metastases in the spleen and liver (Gilbert *et al*, 1995). The significance of this increase in DDC activity and resultant monoamine synthesis by the cancer cells is still unknown (Berry *et al*, 1996), yet it is closely related to the implication of DDC in cancer.

In this study, we investigated the expression of the *DDC* gene in colorectal adenocarcinoma and its prognostic significance. Our study revealed a statistically significant, negative association between *DDC* mRNA expression levels and the histologic tumour grade (*P*=0.011). Colorectal tumours of low histologic grade (I) were more frequently *DDC*-positive, in contrast with malignancies of high grade (II/III). These data seem to indicate that higher *DDC* expression is associated with well-differentiated intestinal tumours.

In accordance with the above-mentioned results, the Kaplan–Meier analysis showed significantly higher DFS and OS time for patients having positive *DDC* mRNA expression (*P*=0.009 and *P*=0.027, respectively). Cox univariate regression analysis showed that *DDC*-positive patients had a significantly lower risk of relapse (∼5 times) and a higher probability of survival (∼4 times). In the Cox multivariate regression model, the levels of *DDC* mRNA were adjusted for patients’ nodal status, Dukes’ stage and histologic tumour grade. When all these non-molecular parameters were included in the multivariate analysis model, *DDC* mRNA expression did not show statistically significant independence as a prognostic factor for DFS or OS of patients with colorectal adenocarcinoma.

In conclusion, this study revealed that higher mRNA expression levels of *DDC* are related with less advanced and/or aggressive tumours. Our results imply that *DDC* mRNA overexpression is linked to favorable prognosis in patients with colorectal adenocarcinoma and may constitute a useful tissue biomarker. Involvement of DDC in apoptosis of colon cancer cells and/or response of *DDC*-positive tumours to chemotherapy are two potential explanations, but further investigation is required to clarify the role of DDC in CRC.

## Figures and Tables

**Table 1:** Distribution of numerical variables of the study in 95 colorectal adenocarcinoma patients

			**Percentiles**		
			**25**	**50**	**75**
**Variable**	**Mean±s.e.^a^**	**Range**	**Median**		
*DDC* in tumours (c**/**Kc^b^)	9.02±1.51	0.04–91.95	0.17	3.09	12.82
Patient age (years)	67.3±1.01	35–88	62.0	68.0	75.0
DFS (months)	36.6±2.63	0.5–79.0	16.7	39.5	52.2
OS (months)	38.1±2.63	0.5–79.0	17.7	42.5	54.2

Abbreviations: DDC=L-DOPA decarboxylase; DFS=disease-free survival; OS=overall survival.

a Standard error of the mean.

b *DDC* mRNA copies per 1000 *GAPDH* mRNA copies.

**Table 2:** Relationships between *DDC* status^a^ and other clinicopathological variables

		**Number of patients (%)**		
**Variable**	**Total**	***DDC* negative^a^**	***DDC* positive^a^**	***P*-value**
*Nodal status*				
Positive	49	35 (71.4)	14 (28.6)	0.63^b^
Negative	41	32 (78.0)	9 (22.0)	
X	5			
				
*Stage* c				
A/B	47	33 (70.2)	14 (29.8)	0.13^b^
C/D	41	35 (85.4)	6 (14.6)	
X	7			
				
*Histologic grade*				
I	6	2 (33.3)	4 (66.7)	0.011^d^
II	70	51 (72.9)	19 (27.1)	
III	11	11 (100)	0 (0.00)	
X	8			

Abbreviations: DDC=L-DOPA decarboxylase; x=status unknown.

a Cut-off point: 12.82 c/Kc, equal to the 75th percentile.

b Calculated by Fisher's exact test.

c Dukes’ staging system.

d Calculated by *χ*^2^-test.

**Table 3:** *DDC* expression and survival of patients with colorectal adenocarcinoma

	**Disease-free survival**			**Overall survival**		
**Variable**	**HR^a^**	**95% CI^b^**	***P*-value**	**HR^a^**	**95% CI^b^**	***P*-value**
*Univariate analysis*						
*DDC*						
Negative	1.00			1.00		
Positive	0.18	0.043–0.77	0.021	0.23	0.053–0.97	0.047
As continuous variable	0.96	0.93–1.00	0.089	0.95	0.90–1.00	0.089
Nodes positive	1.80	0.83–3.89	0.13	2.69	1.11–6.51	0.027
Stage (ordinal)	2.31	1.42–3.73	0.001	2.53	1.48–4.34	0.001
Histologic grade (ordinal)	3.07	1.33–7.05	0.008	3.92	1.57–9.74	0.003
						
*Multivariate analysis* c						
*DDC*						
Negative	1.00			1.00		
Positive	0.37	0.08–1.67	0.19	0.56	0.12–2.67	0.46
Nodes positive	0.76	0.29–2.00	0.58	1.14	0.36–3.58	0.82
Stage (ordinal)	2.31	1.30–4.10	0.004	2.32	1.14–4.74	0.021
Histologic grade (ordinal)	2.13	0.86–5.30	0.103	2.70	0.98–7.41	0.054

Abbreviations: CI=confidence interval; *DDC*=L-DOPA decarboxylase; HR=hazard ratio.

a HR estimated from Cox proportional hazard regression model.

b CI of the estimated HR.

c Multivariate models were adjusted for patients’ nodal status, Dukes’ stage, and histologic tumour grade.
